# The Trend of Utilization of Dental and Oral Health Services Among Children in Iran During 2019–2021: A Cross‐Sectional Study

**DOI:** 10.1002/hsr2.70301

**Published:** 2024-12-29

**Authors:** Somayeh Khoramian Tusi, Behrooz Pouragha, Edris Hasanpoor, Morteza Nazari

**Affiliations:** ^1^ Department of Pediatric Dentistry, School of Dentistry Alborz University of Medical Sciences Karaj Iran; ^2^ Department of Healthcare Services Management, School of Health Alborz University of Medical Sciences Karaj Iran; ^3^ Research Center for Evidence‐Based Health Management Maragheh University of Medical Sciences Maragheh Iran

**Keywords:** dental health, oral health, pediatric dentistry, public health, utilization

## Abstract

**Background and Aims:**

Oral health issues present substantial obstacles for children and teenagers globally, affecting their holistic health. With Iranian children experiencing a high prevalence of dental cavities, understanding their utilization of dental services and the influencing factors is essential. Therefore, this study examined the utilization of dental and oral health services by children aged 6 to 14 at healthcare centers in Karaj, Iran, in 2019–2021.

**Methods:**

This descriptive cross‐sectional study included 361 children between 6 and 14 years old who visited healthcare centers in Karaj for dental services. The researchers developed a questionnaire to collect data. Data analysis was conducted using SPSS‐21 software, Smirnov–Kolmogorov, and Spearman's correlation test.

**Results:**

Analysis of the data indicated that the utilization of dental and oral health services had significant correlations with the place of residence (*p* < 0.001), the father's education (*p* < 0.05), the number of children in the family (*p* < 0.005), and insurance coverage (*p* < 0.05). However, household income and the mother's education had no significant relationship with the utilization of dental and oral health services for the children in healthcare centers (*p* > 0.05).

**Conclusions:**

Advocate for policies promoting oral health equity, addressing dental service access disparities, and supporting preventive measures in underserved communities, provision of insurance coverage for dental services can lead to improving the oral health of children.

## 1 Introduction

Health is a human right and every person has the right to demand it. The main purpose of healthcare service delivery is to ensure the health of people in the community. Inadequacy in the provision and distribution of medical services around the world, especially in developing countries, requires establishing primary health care providers [1]. To this end, the primary healthcare network in Iran has expanded significantly to provide healthcare services including dental and oral services to people. The main part of these dental services includes services provided to children aged 6 to 14, such as health education, preventive services, restoration of deciduous and permanent teeth, treatment of the live pulp of deciduous teeth, extraction of deciduous and permanent decayed teeth, and so forth [2].

The healthcare system's primary goal is to ensure the comprehensive provision of oral health services through efficient planning and equitable resource distribution. This objective is realized when individuals have the capability and willingness to access and utilize these services. The physical availability of services to the population is a key factor influencing access. Moreover, factors such as cost, quality, timeliness of care, and public awareness of oral health services are significant determinants of the utilization of these services by individuals [3].

Efficient healthcare systems rely on the delivery of desirable services to fulfill their mission of providing health to the community. Patient feedback is a crucial tool for evaluating the quality of care and services, emphasizing the importance of considering patients' opinions in healthcare planning and evaluation. Recognizing clients' needs and monitoring the utilization of dental services are integral components of effective healthcare system planning [4].

Optimal oral health is an integral part of good public health. Many oral health problems, including tooth decay, begin in childhood and can be prevented with early dental care. Although the provision of good oral health is beyond just having healthy teeth, many children do not have adequate oral and general health due to active and uncontrolled dental caries. According to the US Federal Agency Report on Oral Health released in May 2000, tooth decay is the most common chronic childhood disease, and all experts agreed generally that dental caries is an infectious and contagious disease and should be given special attention [5].

With adequate training health professionals can develop their domain of practice to provide oral health education, risk assessments and dental referrals. Such interventions could provide a maintained improvement in oral health outcomes of children [6, 7].

Educational initiatives have emerged as a crucial strategy to prevent childhood caries. These initiatives aim to empower children and their caregivers with knowledge about oral hygiene practices, dietary choices, and the importance of regular dental visits. By fostering an understanding of the causes and prevention of dental caries, these programs seek to instill lifelong habits that promote oral health and reduce the incidence of cavities [2, 3, 4].

According to previous studies, by the end of infancy, 50% of children have one or more decayed baby teeth, but the importance of these teeth should not be neglected, because healthy teeth in childhood play an important role in the growth of healthy permanent teeth, healthy nutrition, and the physical attractiveness of a person [6, 7].

Furthermore, factors contributing to tooth decay include malnutrition, genetic susceptibility, poor oral health practices, dietary habits, decay‐causing bacteria like streptococci, lack of fluoride and vitamin D, excessive sugar consumption, and prolonged milk feeding, Additional factors such as age, gender, and geographical location also play a role in the development of tooth decay [8].

In addition, factors affecting tooth decay include: malnutrition, genetic susceptibility, bad oral hygiene practices, dietary habits, caries bacteria such as streptococcus, lack of fluoride and vitamin D, excessive sugar consumption and long‐term nutrition. Other factors such as age, gender and geographic location also play a role in causing tooth decay [8].

The World Health Organization (WHO) has identified dental caries in childhood as a global problem with a prevalence of 60% to 90% [9]. Data from European countries indicated that 61% of children aged 6 to 12 have at least one decayed tooth. The wide prevalence of caries in all social classes can impose a large financial burden on society [10].

In Iran, the average DMF index (Decayed, Missed, and Filled) in children is below the global average, highlighting the need for targeted efforts to improve access to dental services, particularly in the public sector [11]. Recent efforts by both public and private sectors within the healthcare system have highlighted the critical importance of not only a global focus but also a detailed assessment of public access to healthcare services [12, 13].

Since numerous factors are affecting the use of health services, it is essential to identify and adjust the factors effective in improving the quantity of using services. These factors can be socioeconomic factors or the parameters related to the health care delivery system such as access to health care resources, public insurance coverage, etc [12, 14]. Accordingly, as no study has addressed the coverage of dental and oral health services in Karaj, this study addressed the utilization of these services by children aged 6 to 14 years referring to healthcare centers in Karaj.

## 2 Materials and Methods

This descriptive cross‐sectional study was conducted on children aged 6 to 14 years covered by healthcare centers providing dental services in Karaj who referred to these centers from November 2019 to late March 2020. The sample size in this study was estimated using Cochran's formula as follows:

$$n=\frac{\frac{z^{2}\mathrm{pq}}{d^{2}}}{1+\frac{1}{N}\;\left[\frac{z^{2}\mathrm{pq}}{d^{2}}-1\right]}$$

Where *p* and *q* are the success and failure ratios, which are taken as equal to 0.5, *Z* is the value of the normal variable of the standard unit, which is 1.96 at the 95% confidence level, *d* is the error value equal to 0.05, and *N* represents the size of the target population.

The research population included 6000 children aged 6 to 14 years covered by healthcare centers providing dental services. Given the research population and following Cochran's formula, the sample size was calculated with an error value of 0.05 equal to 361 persons. The research setting was all healthcare centers providing dental services in the city of Karaj and the inclusion criteria were children aged 6 to 14 years and the willingness to participate in the study.

A researcher‐made questionnaire was used to collect data. It consisted of three sections and 36 items. The first section included 21 items that assessed the participants' demographic variables, including gender, citizenship, age, place of residence, length of residence in Karaj, guardianship, parental job and income, family expenses, parents' education, number of family members, number of children in the family, birth order, household income, health insurance status, housing ownership, owning a personal car, and family access to the Internet. The second section contained seven items to measure the access to dental centers, and the burden and frequency of referring to healthcare centers. Finally, the third section contained eight items that assessed the participants' oral health and the quantity and types of dental services received in the past.

To assess the content validity of the questionnaire, it was reviewed and validated by experts in the field and was administered to the participants after making required revisions in its content based on the experts' opinions. The reliability of the questionnaire was assessed using Cronbach's *α*. Before conducting the study, this questionnaire was administered to a pilot sample of 15 persons and the related Cronbach's *α* values for the three subscales of the questionnaire were estimated. The reliability of the questionnaire was between 0.77 and 0.89 (Demographic variables [*r* = 0.89], access and referral [*r* = 0.79] and dental and oral services received [0.77]).

The researcher completed the questionnaires by reaching out to healthcare centers in both the Eastern and Western regions of Karaj, such as Turkian, 24 Mehr, Anbari, Rastravesh, Imam Hassan Mojtaba (AS), Shohaday‐e Aq Tappeh, Avini, Hajizadeh, Hesarak Bala, and Zafar healthcare centers. After explaining the study's objectives to the clients, the researcher conducted semi‐structured interviews with the parents of the children and filled out the questionnaires based on their responses. Clients who were uninterested in participating were excluded from the study. If necessary, additional information was gathered from the clients' medical records.

The participants' responses were codified and the resulting data were entered into SPSS‐21 software and analyzed. Descriptive statistics (mean, frequency, and percentage) were calculated with all survey items and total scores, as well as demographic data. All available data were used for analysis, and no participants were removed from the analysis due to missing data. Since the normality of the data was not confirmed, Spearman's correlation test was used. Spearman's correlation test was used to assess the relationship between the research variables. All tests are considered significant at the 0.05 level.

## 3 Results

The participants were 361 children aged 6 to 14 years. The data showed that 41.6% of the participants were boys and 58.4% were girls. Moreover, 47.9% of the children were aged 6 to 10 years and 52.1% were aged 11 to 14 years. In addition, 11.9% of the families of the children lived on the outskirts of the city and 88.1% lived in the city center. The results also indicated that 8.3% of fathers in this study had high school and lower education, 52.6% had a diploma, and 39.1% had higher education. Furthermore, 11.4% of the mothers in the sample had high school and lower education, 58.2% had a diploma, and 30.5% had higher education. According to the findings, 17.5% of the families of the children had an income of lower than 2 million Tomans, 49.9% have an income of between 2 and 4 million, and 32.7% have an income of more than 4 million Tomans.

As can be seen in Table 1, 54% of families had one child aged less than 14 years, 36% of the families had two children aged less than 14 years, 7% had three children aged less than 14, and 1% of the families had four and more children aged less than 14 years.

**Table 1:** The frequency of children referring to dentistry.

Number of visits	0	1	2	3	4 and more	Mean	SD	Maximum	Minimum	Total
Frequency	100	75	85	81	20	2.57	1.25	5	1	361
Percentage	27.7%	20.8%	23.5%	22.4%	5.5%					100

The data in Table 2 suggested that 33% of the participants had health insurance, 53.5% had social security insurance, 3.9% used other types of insurance and 9.7% of the participants did not have any insurance coverage.

**Table 2:** The distribution of the children by age and insurance.

Variables	Frequency	Percentage
Number of children		
1	224	54
2	130	36
3	27	7
4 and more	7	1
Total	361	100
Type of insurance		
Treatment	119	33
Social security	193	53.5
Other	14	3.9
No insurance	35	9.7
Total	361	100

### 3.1 Data Normality Test

The Smirnov–Kolmogorov test was run to check the normality of the distribution of variables. As the significance level for the data for all variables is less than 0.05, it can be concluded that none of the variables follow the normal distribution. Thus, nonparametric methods were used to analyze the data.

To evaluate the dental service utilization provided by the healthcare centers for children aged 6 to 14, the average number of visits to healthcare centers during 1 year was examined. The data in Table 1 reveal that the average number of children referred to the dentist was 2.57, indicating that the average number of visits to the dentist varies from two to three times. Moreover, the majority of the children used dental services four times or more and only a small percentage of the children never referred to a dentist. Moreover, 100 children did not go to the dentist at all, 75 children went to the dentist once, 85 children twice, 81 children three times, and 20 children went to the dentist more than four times.

Spearman's rank correlation test (Table 3) was run to investigate the effect of the place residence on the utilization of oral health services for the children. Given that the significance level of the test for all relationships is less than 0.05, it can be suggested that the utilization of dental services is directly associated with the participants' place of residence of people, indicating that the utilization of dental services in the suburbs was less than the services offered in the city center (*p* < 0.05). On the other hand, the utilization of dental services was negatively correlated with the distance from the dental centers and the time that must be spent on travel. Thus, the greater the distance from the dental centers, the fewer children go to healthcare centers for dental services.

**Table 3:** Spearman's correlation test to investigate the relationship of the place of residence and physical and temporal distance with the utilization of dental health services.

Variable	Service utilization	Place of residence	The nearest temporal distance to the healthcare center	The nearest physical distance to the healthcare center
**Service utilization**	1	*r* = 0.196 *p* = 0.000	*r* = −0.205 *p* = 0.000	*r* = −0.225 *p* = 0.000
**Place of residence**	—	1	*r* = −0.336 *p* = 0.000	*r* = −0.347 *p* = 0.0
**The nearest temporal distance to the healthcare center**	—	—	1	*r* = 0.923 *p* = 0.0
**The nearest physical distance to the healthcare center**	—	—	—	1

Given that the significance level of Spearman's test for the relationship between the oral service utilization and the father's education (Table 4) is less than 0.05, it can be concluded that the father's education has a significant positive relationship with the utilization of oral health services for the children (*p* < 0.05). Thus, an increase in the father's education can positively affect the utilization of dental services for children. However, the results indicated that the mothers' education has no significant relationship with the utilization of dental services (*p* > 0.05).

**Table 4:** Spearman's correlation test to investigate the relationship between parental education and utilization of dental health services.

Variable	Service utilization	Father's education	Mother's education	Household income	Number of children
Service utilization	1	*r* = 0.119 *p* = 0.024	*r* = 0.532 *p* = 0.53	*r* = 0.06 *p* = 0.199	*r* = −0.11 *p* = 0.037

Given that the significance level of Spearman's test for the relationship between the oral service utilization and the household income (Table 4) is greater than 0.05, it can be concluded that the household income has no significant positive relationship with the utilization of oral health services for the children (*p* > 0.05).

Following the data presented in Table 4, it can be argued that the number of children aged 6 to 14 has a significant negative relationship with the utilization of oral health services for the children (*p* < 0.05). Thus, an increase in the number of children aged 6 to 14 reduces the utilization of dental services for children.

Given that the significance level of Spearman's test for the relationship between the oral service utilization and the type of treatment insurance (Table 5) is less than 0.05, it can be suggested that having treatment insurance has a significant positive relationship with the utilization of oral health services for the children (*p* < 0.05). Furthermore, having supplemental dental insurance has a significant positive impact on the utilization of oral health services for the children.

**Table 5:** Spearman's correlation test to investigate the relationship between the type of treatment insurance and utilization of dental health services.

Variable	Service utilization	Treatment insurance	Supplemental dental insurance	Type of insurance
Service utilization	1	*r* = 0.395 *p* = 0.023	*r* = 0.393 *p* = 0.027	*r* = 0.231 *p* = 0.0
Treatment insurance	—	1	*r* = 0.483 *p* = 0.0	*r* = 0.264 *p* = 0.012
Supplemental dental insurance	—	—	1	*r* = 0.314 *p* = 0.0
Type of insurance	—	—	—	1

## 4 Discussion

Tooth decay is one of the major health problems faced by children and adolescents around the world and has a serious impact on the quality of life of children with a growing prevalence in many countries [15]. In Iran, the average DMF index in children is weak compared to the global average and requires special attention, practical decisions, facilitating access and use of dental services, especially public sector services [11].

The high prevalence of dental caries and the high DMFT (Decayed, missing, and filled teeth) index in Iranian children warrants the study of the utilization of dental services and also the recognition of effective factors and their modification. These factors can be socioeconomic factors or factors related to the health care delivery system such as access to health care resources, public insurance coverage, and so forth [12, 14].

The results of the present study indicated that the coverage/provision of dental services is directly associated with the client's place of residence; this means that the utilization of dental services in the suburbs is less than in the city center. Furthermore, the utilization of dental services is inversely associated with the distance from the dental centers and the distance that must be traveled. Thus, the greater the distance from the dental centers, the less likely are children to go to healthcare centers for dental services. Because a person can use health‐related services when the services are available. Geographical access and temporal access have been highlighted in most studies as factors influencing the use of services. For instance, in their study in Brazil, Curi et al. reported that dentists' availability was one of the factors affecting the utilization of dental health services by the pediatric population [16].

Moreover, Maftoon showed a significant difference between the demand load and referrals for medical services in different districts of Tehran, so the highest frequency of referrals was observed in downtown Tehran [17]. In another study in Gonbad‐e Kavus, Gholami et al. reported a significant relationship between the area of residence and the number of referrals to dentistry [18]. Heidarnia et al. also stated that the long‐distance to rural healthcare centers adversely affect the utilization of health services in Damavand city [19].

The data in the present study indicated that the father's education has a significant positive relationship with the utilization of dental services. However, the mother's education does not affect the extent to which children utilize dental services. Demographic characteristics, including education, have always been an important factor in accessing health‐related services.

A study by Barasuol et al. in Brazil showed that the lower levels of education of the head of the household were associated with a significant reduction in the utilization of oral health services [20]. Furthermore, in their study in Brazil, Curi et al. reported that factors related to health care providers such as education and health literacy were among the factors affecting the utilization of dental services [16].

Another study by Rezaei in western Iran showed that the use of dental health services among households in the study area was influenced by several social and economic factors, including parents' university education [21]. Moreover, in their study in the USA, Finlayson et al. found that children with educated caregivers had more visits to dentistry [22].

Ebadifard et al. showed that as the level of parental education increases, so does the demand for health‐related services [23]. In line with the findings of the present study, a study by Leinsalu et al. in Estonia and Lithuania showed that educated people had more dental visits. Moreover, Gholami et al. showed a significant relationship between the level of education of the head of the household and referral to dentistry. As the level of education of the head of the household increases, the probability of using dental services increases [18].

The findings of the present study indicated that household income does not have a significant effect on children's utilization of oral health services in healthcare centers. This finding was in line with the results reported by Barasuol et al. but contrary to the observations made by Rezaei and Ebadifard et al. Many studies have highlighted household income as one of the most important factors affecting the utilization of health‐related services. In their study in Isfahan province, Ebadifard et al. showed that total household income has a positive and significant effect on health‐related services [24].

Gholami et al. reported a positive significant relationship between income and referring to dentistry [18]. The reason for this discrepancy is probably the provision of low‐cost and in some cases very cheap and free dental services by healthcare centers. Thus, using these services does not cost the family much.

In a systematic review, Hooley et al. found that demographic characteristics such as the number of children in a family and the birth order affect children's oral health [25]. The findings of the present study indicated that the more children in the family, the lower the rate of utilization of dental health services. However, the order of childbirth did not have a significant effect on the utilization of dental health services. Furthermore, Ebadifard et al. showed that the number of children in the family has a positive and significant effect on the use of health services [24].

Following the findings of the present study, Rezaei and Ebadifard stated that having treatment insurance has a positive and significant relationship with the utilization of dental health services [21, 25]. Accordingly, having treatment insurance is directly associated with the utilization of dental health services. On the other hand, having supplementary dental insurance has a positive and significant effect on the utilization of dental health services by children. Gholami et al. also suggested that people with insurance coverage had the highest number of referrals to dental services, while people without insurance coverage had the lowest number of referrals to these services [18].

## 5 Conclusions

In conclusion, the analysis of factors influencing the utilization of oral health services for children aged 6 to 14 highlights the importance of parental education, insurance coverage, family size, and place of residence in shaping access to dental care. While higher paternal education and insurance coverage were associated with increased service utilization, larger family sizes, and suburban residences were linked to lower utilization rates.

These findings provide valuable insights for designing targeted interventions to enhance dental service access and utilization among children in diverse demographic settings, such as Community Outreach Programs: Implement initiatives to increase awareness about the importance of oral health and available services, particularly targeting families in suburban areas, Education Campaigns: Provide educational resources for parents, emphasizing the benefits of regular dental check‐ups and good oral hygiene practices. School‐Based Programs: Collaborate with schools to offer preventive dental services and screenings, fostering a culture of oral health awareness among students and parents. Financial Assistance: Develop programs to support families with limited income or without insurance coverage to access affordable dental care for their children.

By implementing targeted strategies that address the specific needs and challenges identified in the study, stakeholders can work towards improving the utilization of dental health services for children aged 6 to 14, ultimately promoting better oral health outcomes and overall well‐being.

## Author Contributions

**Somayeh Khoramian Tusi:** conceptualization, data curation, formal analysis, funding acquisition, investigation, resources. **Behrooz Pouragha:** funding acquisition, project administration, software, validation, visualization. **Edris Hasanpoor:** investigation, validation, visualization, writing–original draft, writing–review and editing. **Morteza Nazari:** data curation, formal analysis, investigation.

## Conflicts of Interest

The authors declare no conflicts of interest.

## Transparency Statement

The lead author Morteza Nazari affirms that this manuscript is an honest, accurate, and transparent account of the study being reported; that no important aspects of the study have been omitted; and that any discrepancies from the study as planned (and, if relevant, registered) have been explained.

## Data Availability

The data that support the findings of this study are available from the corresponding author upon reasonable request.
